# Neuroprotective Effect of Quercetin and Memantine against AlCl_3_-Induced Neurotoxicity in Albino Wistar Rats

**DOI:** 10.3390/molecules28010417

**Published:** 2023-01-03

**Authors:** Ratnakar Jadhav, Yogesh A. Kulkarni

**Affiliations:** Shobhaben Pratapbhai Patel School of Pharmacy & Technology Management, SVKM’s NMIMS, V.L. Mehta Road, Vile Parle (West), Mumbai 400056, India

**Keywords:** quercetin, memantine, oxidative stress, amyloid beta, acetylcholinesterase, BDNF, neurotoxicity, Alzheimer’s disease

## Abstract

Recent evidences indicate that there is a substantial increase in worldwide cases of dementia. Alzheimer’s disease is the leading cause of dementia and may contribute to 60–70% of cases. Quercetin is a unique bioflavonoid that has numerous therapeutic benefits such as anti-allergy, anti-ulcer, anti-inflammatory, anti-hypertensive, anti-cancer, immuno-modulatory, anti-infective, antioxidant, acetylcholinesterase inhibitory activity, neuroprotective effects, etc. In the present study, we evaluated the neuroprotective effect of orally administered quercetin with memantine in albino Wistar rats after inducing neurotoxicity through AlCl_3_ (100 mg/kg, p.o.). Chronic administration of AlCl_3_ resulted in poor retention of memory and significant oxidative damage. Various behavioral parameters, such as locomotor activity, Morris water maze, elevated plus maze, and passive avoidance test, were assessed on days 21 and 42 of the study. The animals were euthanatized following the completion of the last behavioral assessment. Various oxidative stress parameters were assessed to know the extent of oxidative damage to brain tissue. Quercetin with memantine has shown significant improvement in behavioral studies, inhibition of AChE activity, and reduction in oxidative stress parameters. Histopathological studies assessed for cortex and hippocampus using hematoxylin and eosin (H&E), and Congo red stain demonstrated a reduction in amyloid-β plaque formation after treatment of quercetin with memantine. Immunohistochemistry showed that quercetin with memantine treatment also improved the expression of brain-derived neurotrophic factor (BDNF) and inhibited amyloid-β plaque formation. The present study results demonstrated protective effects of treatment of quercetin with memantine in the neurotoxicity linked to aluminum chloride in albino Wistar rats.

## 1. Introduction

As per recent reports from WHO, around 50 million people have dementia, and Alzheimer’s disease (AD) may contribute to about 60–70% of these cases [1]. With the increase in the world’s aging population prevalence of AD is going to be soaring in the coming decades. It is projected that by 2050 the population of the world over 60 years of age shall be almost 2 billion [2]. In 2005, Alzheimer’s Disease International (ADI) appointed a panel of experts to evaluate epidemiological data. The panel estimated that every year, 4.6 million new cases of AD will be added, and numbers will be doubled every 20 years, reaching 81.1 million by 2040 [3].

The aging population puts imperious demands on countries’ healthcare system and most of the countries are ill-prepared for such demands. From 2000 to 2018, there was a 146.2% increase in the deaths reported from AD; on the contrary, deaths resulting from stroke, HIV, and heart disease decreased. 

AD is a neurodegenerative disorder characterized by cognitive dysfunction and behavior impairment [4]. The neurodegenerative process is characterized by an impairment of the synapses with degeneration of the axons [5]. The key pathological hallmarks of AD are noxious extracellular deposits of β-amyloid plaques and intracellular aggregates of hyperphosphorylated tau proteins called neurofibrillary tangles (NFT) [6]. 

In recent times, the understanding of Alzheimer’s disease progression and pathogenesis has been a subject of intense debate and discussion among the scientific community for aiding the development of pharmacotherapy for the management and treatment of AD. Current treatment options for AD propose two strategies; (1) symptomatic and (2) disease-modifying approaches [7]. At present, approved pharmacological treatment for AD includes cholinesterase inhibitors (ChEIs) and N-methyl-d-aspartate (NMDA) glutamate receptor antagonists. Moreover, in 2014, FDA also approved a combination of memantine and ChEIs for patients with moderate-to-severe AD [8].

Memantine, (1-amino-3, 5-dimethyl-adamantane), is a non-competitive, N-methyl-D-aspartate (NMDA) receptor antagonist. Memantine has been approved by FDA for the treatment of moderate to advanced symptoms of AD. Memantine exerts its pharmacological effect through its moderate affinity to NMDA receptor. Memantine inhibits the effects of elevated levels of glutamate that leads to neuronal dysfunction through its Ca^2+^ influx [9]. 

Although present pharmacotherapy does not cure or inverse the neurodegeneration of AD, the treatment improves memory loss and cognitive functions. The cholinesterase inhibitors have been reported with serious adverse events include gastrointestinal complaints such as nausea, vomiting, loss of appetite, headache, insomnia, and dizziness occurring in 20% of patients. Further other systemic effects related to cholinergic activity include urinary urgency, bradycardia, and syncope. Because of this, close monitoring of the patient and dose titration has been recommended [8,10,11].

Therefore, it is fundamental to pursue new strategies for the development of pharmaco-therapy for AD. A variety of natural compounds were reported with neuroprotective effects, which has increased the interest of the researchers and pharmaceutical industry. The inclination of the scientists toward the natural product is increasing day by day not only due to their effectiveness but also due to safety profile. Natural compounds are also receiving special attention for the development of “multiple-targets lead” (MTD) for the treatment of AD [12].

Flavonoids have demonstrated a wide spectrum of health benefits and are part of various nutraceuticals, pharmaceuticals, and cosmetic products [13]. Quercetin is a unique bioflavonoid ubiquitously found in onion, broccoli, apples, berries, tea, and red wines. Quercetin has been reported with a wide range of therapeutic activities, such as antioxidant, anti-inflammatory, anti-amyloidogenic, and neuroprotective activities [14,15,16,17]. The ability of quercetin to inhibit xanthine oxidase and lipid peroxidation or its oxygen-scavenging activity exerts its neuroprotective activity [18]. Further, quercetin administration in 3xTg-AD mice has shown reversal of paired helical filaments (PTH), β-amyloid (βA) 1–40, and βA 1–42 levels due to inhibition of phosphorylation of AT-8 tau in the brain [19]. Moreover, quercetin has been reported to regulate the Akt/PKB and ERK1/2 signaling pathway by impeding the activity of PI3K, resulting in its neurotropic effect [20].

In this study, we evaluated the effect of quercetin with memantine on the reversal of neurotoxicity induced by AlCl_3_ in albino Wistar rats.

## 2. Results

### 2.1. Behavioral Assessment

#### 2.1.1. Actophotometer

**Figure 1:**
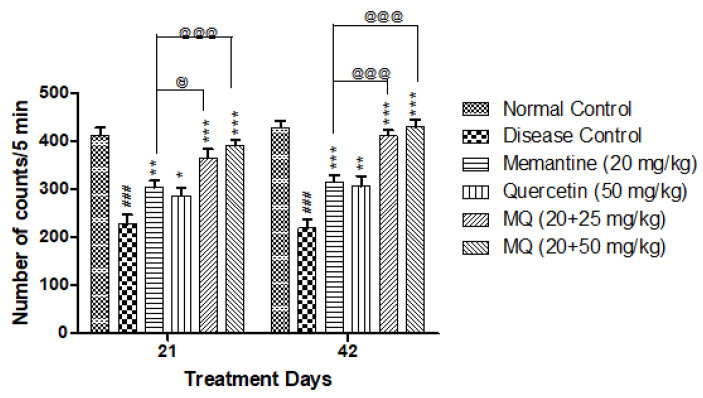
Effect of quercetin and quercetin with memantine on locomotor activity in AlCl_3_-induced neurotoxicity in rats. Data are expressed as mean ± SEM (*n* = 6), ### *p* < 0.001 when compared with the normal control group, * *p* < 0.05, ** *p* < 0.01, *** *p* < 0.001 when compared with the disease control group, and @ *p* < 0.05, @@@ *p* < 0.001 when compared with the memantine treatment group; AlCl_3_: aluminum chloride, SEM: standard error of mean.

It was observed that the locomotor activity was significantly decreased in the disease control group treated with AlCl_3_ when compared to the normal control group. An administration of quercetin with memantine at a dose of 20 + 25 and 20 + 50 mg/kg significantly improved locomotor activity on day 21 and day 42 (*p* < 0.001) when compared with the disease control group. The administration of quercetin with memantine at a dose of 20 + 25 mg/kg showed improvement on day 21 (*p* < 0.05) and day 42 (*p* < 0.001) when compared with the memantine treatment group. The group treated with 20 + 50 mg/kg showed significant improvement in locomotor activity on both days 21 and 42 at a significance level of *p* < 0.001 when compared with the group treated with memantine (Figure 1).

#### 2.1.2. Morris Water Maze (MWM)

**Figure 2:**
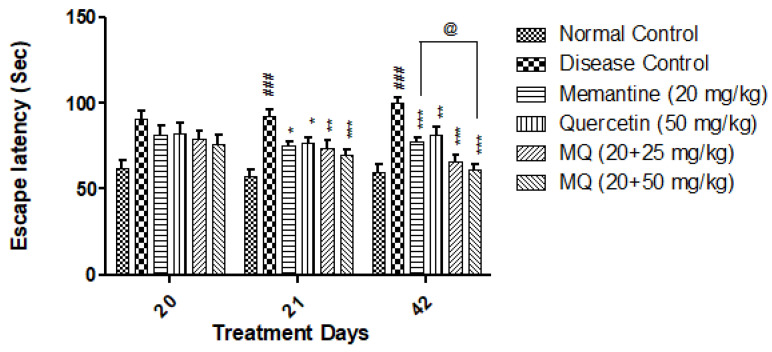
Effect of quercetin and quercetin with memantine on Morris water maze test in AlCl_3_-induced neurotoxicity in rats. Data are expressed as mean ± SEM (*n* = 6), ### *p* < 0.001 when compared with the normal control group, * *p* < 0.05, ** *p* < 0.01, *** *p* < 0.001 when compared with the disease control group, and @ *p* < 0.05 when compared with the memantine treatment group; AlCl_3_: aluminum chloride; SEM: standard error of mean.

In the Morris water maze test, a significant increase (*p* < 0.001) in escape latency was seen in the AlCl_3_-treated group when compared with the normal control group on days 21 and 42. The administration of quercetin with memantine at a dose of 20 + 25 mg/kg significantly decreased escape latency on day 21 (*p* < 0.01) and day 42 (*p* < 0.001) when compared with the disease control group. Moreover, a dose of 20 + 50 mg/kg quercetin with memantine significantly decreased (*p* < 0.001) escape latency on both days 21 and 42. Further, at this dose, an improvement in the retention performance of the spatial navigation task was also observed at a significance level of *p* < 0.05 on day 42 when compared with the group treated with memantine alone (Figure 2).

#### 2.1.3. Elevated Plus Maze (EPM)

**Figure 3:**
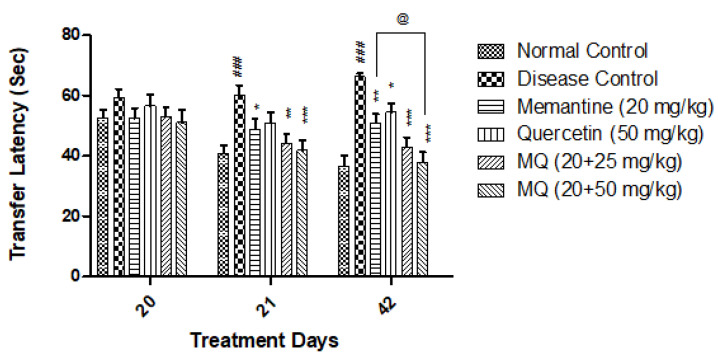
Effect of quercetin and quercetin with memantine on transfer latency in AlCl_3_-induced neurotoxicity in rats. Data are expressed as mean ± SEM (*n* = 6), ### *p* < 0.001 when compared with the normal control group, * *p* < 0.05, ** *p* < 0.01, *** *p* < 0.001 when compared with the disease control group, and @ *p* < 0.05 when compared with the memantine treatment group. AlCl_3_: aluminum chloride; SEM: standard error of mean.

In the elevated plus maze test, a significant increase in transfer latency on day 21 and day 42 has been seen in the AlCl_3_-treated group when compared to the normal control group. Further, administration of quercetin with memantine at a dose of 20 + 25 mg/kg significantly decreased transfer latency on day 21 (*p* < 0.01) and day 42 (*p* < 0.001) when compared with the disease control group. Moreover, quercetin with memantine at a dose of 20 + 50 mg/kg significantly (*p* < 0.001) decreased the escape latency on both days 21 and 42. On day 42, significant decrease in transfer latency was observed in quercetin with memantine treated group (20 + 25 mg/kg, *p* < 0.05) when compared with the memantine-treatment group (Figure 3).

#### 2.1.4. Passive Avoidance (PA)

In the passive avoidance test, the escape latency (EL) was measured on day 20, and the retention latency (RL) was measured on the 21st and 42nd days, respectively.

**Figure 4:**
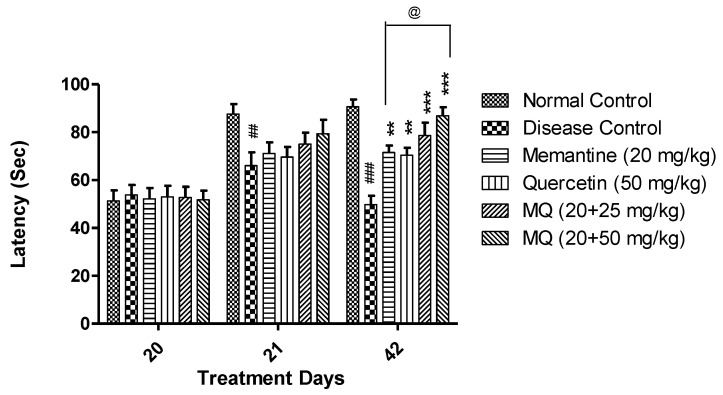
Effect of quercetin and quercetin with memantine on escape latency in AlCl_3_-induced neurotoxicity in rats. Data are expressed as mean ± SEM (*n* = 6), ## *p* < 0.01, ### *p* < 0.001 when compared with the normal control group, ** *p* < 0.01, *** *p* < 0.001 when compared with the disease control group, and @ *p* < 0.05 with the memantine-treatment group. AlCl_3_: aluminum chloride; SEM: standard error of mean.

A significant decrease in retention latency was observed in AlCl_3-_treated group when compared to the normal control group on both days 21 and 42. The animals treated with memantine 20 mg/kg and quercetin 50 mg/kg showed significant increase in retention latency on day 42 (*p* < 0.01); however, administration of quercetin with memantine at a dose of 20 + 25 and 20 + 50 mg/kg showed significant improvement in retention latency on day 42 (*p* < 0.001). Further, treatment with 20 + 50 mg/kg dose showed significant improvement in retention latency (*p* < 0.05) when compared with the memantine-treatment group (Figure 4).

### 2.2. Oxidative Stress Parameters

#### 2.2.1. Assessment of Oxidative Stress Parameters in Cortex

The AlCl_3-_treated group showed a significant increase in MDA levels (*p* < 0.001) and a significant decrease in levels of SOD, CAT, and GSH (*p* < 0.001) in the cortex when compared with normal control group. MDA levels were significantly reduced in the animals treated with quercetin with memantine at a dose of 20 + 25 mg/kg and 20 + 50 mg/kg (*p* < 0.001) when compared with the disease control group.

Quercetin with memantine treatment at a dose of 20 + 50 mg/kg showed significant improvement in CAT and GSH levels (*p* < 0.001) also in SOD levels (*p* < 0.01) when compared with disease control group (Table 1).

#### 2.2.2. Assessment of Oxidative Stress Parameters in Hippocampus

Animals treated with AlCl_3_ showed a significant increase in MDA level (p < 0.001) and a significant decrease in level SOD, CAT, and GSH (*p* < 0.001) in the hippocampus when compared with normal control animals. MDA levels were reduced in quercetin with memantine treatment at a dose of 20 + 25 mg and 20 + 50 mg/kg (*p* < 0.001) when compared with the disease control group. The animals treated with 20 + 25 mg/kg significantly improved SOD and GSH levels (*p* < 0.001) and CAT (*p* < 0.01) when compared with the disease control group. The dose of 20 + 50 mg/kg treatment also showed significant improvement in SOD, CAT, and GSH levels (*p* < 0.001) when compared with the disease control group (Table 2).

### 2.3. Acetylcholinesterase Assay (AChE)

**Figure 5:**
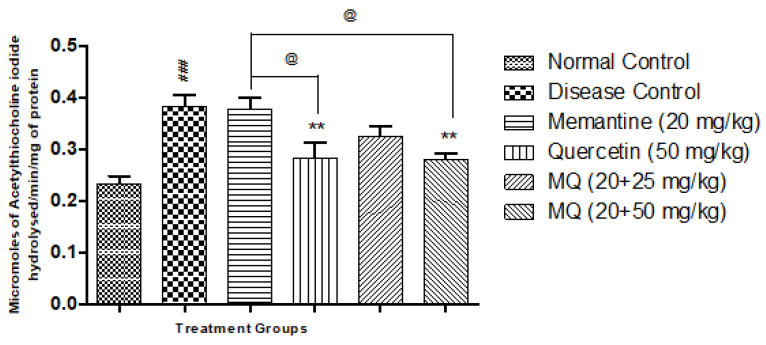
Effect of quercetin and quercetin with memantine on acetylcholine esterase (AChE) in cortex tissue in AlCl_3_-induced neurotoxicity in rats. Data expressed as mean ± SEM (n = 6), ### *p* < 0.001 when compared with the normal control group, ** *p* < 0.01 when compared with the disease control group, and @ *p* < 0.05 when compared with the memantine-treatment group. AlCl_3_, aluminum chloride; SEM, standard error of mean.

**Figure 6:**
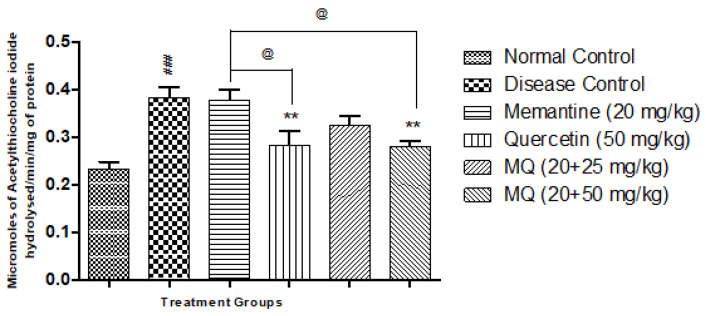
Effect of quercetin and quercetin with memantine on acetylcholine esterase (AChE) in hippocampus tissue in AlCl_3_-induced neurotoxicity in rats. Data are expressed as mean ± SEM (n = 6), ### *p* < 0.001 when compared with normal control, ** *p* < 0.01 when compared with the disease control group, and @ *p* < 0.05 when compared with the memantine-treatment group. AlCl_3_, aluminum chloride; SEM, standard error of mean.

AlCl_3-_treated animals showed a significant increase in AChE activity (*p* < 0.001) in the cortex (Figure 5) and hippocampus (Figure 6) when compared with normal control animals. Treatment with quercetin at a dose of 50 mg/kg and quercetin administered with memantine at a dose of 20 + 50 mg/kg showed significant decrease in AChE activity produced by AlCl_3_ treatment (*p* < 0.01) when compared with disease control animals. Treatment with quercetin with memantine at a dose of 20 + 50 mg/kg showed significantdecrease in AChE activity (*p* < 0.05) when compared with the memantine-treated group in both the cortex and hippocampus (Figure 5).

### 2.4. Histopathological Studies:

#### 2.4.1. Hematoxylin and Eosin

**Figure 7:**
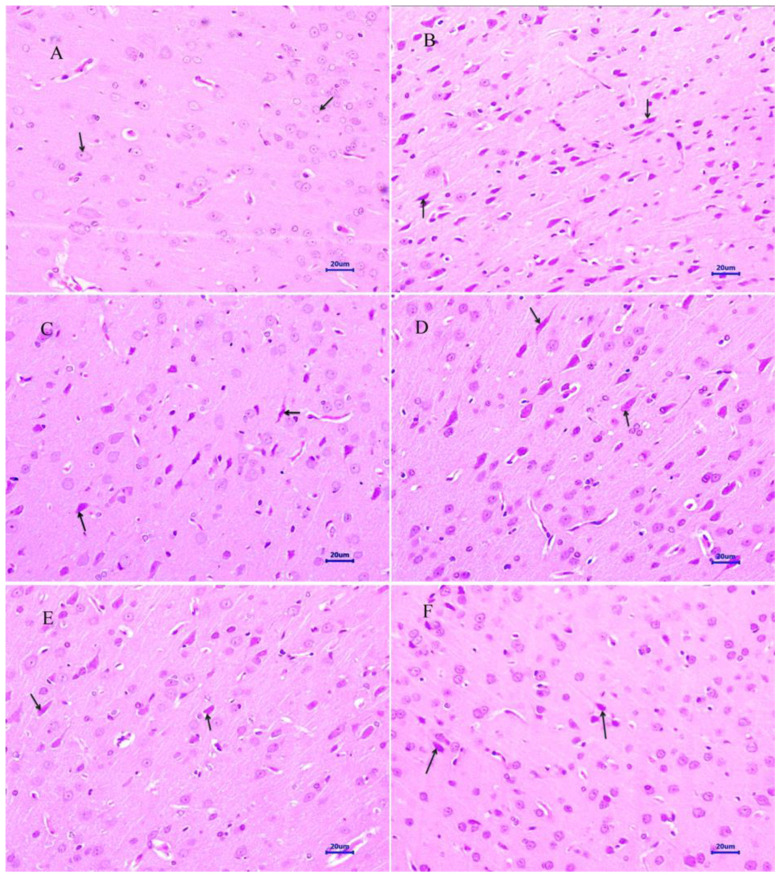
Effect of quercetin and quercetin administered with memantine on H&E-stained cortex tissue (400×). (**A**): Normal control, (**B**): Disease control, (**C**): Memantine (20 mg/kg), (**D**): Quercetin (50 mg/kg), (**E**): Memantine + quercetin (20 + 25 mg/kg), (**F**): Memantine + quercetin (20 + 50 mg/kg).

**Figure 8:**
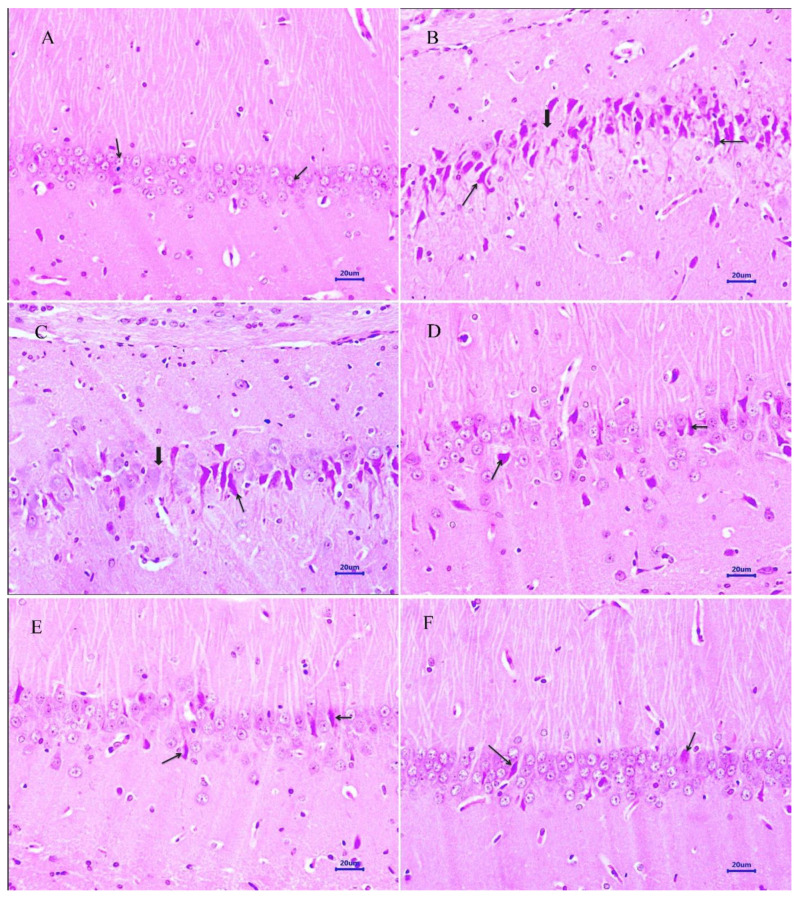
Effect of quercetin and quercetin administered with memantine on H&E-stained hippocampus tissue (400×). (**A**): Normal control, (**B**): Disease control, (**C**): Memantine (20 mg/kg), (**D**): Quercetin (50 mg/kg), (**E**): Memantine + quercetin (20 + 25 mg/kg), (**F**): Memantine + quercetin (20 + 50 mg/kg).

The histopathological assessment of the cortex and hippocampus was performed after staining with hematoxylin and eosin. Microscopic examination of the cortex and hippocampus (Figure 7A and Figure 8A) from normal control group did not show any lesion of pathological significance. The disease control group showed various histopathological changes such as multifocal moderate neuronal degeneration and focal to a multifocal mild-to-moderate reduced layer of neuronal cells in the cortex and hippocampus (Figure 7B and Figure 8B). Treatment with memantine at a dose of 20 mg/kg showed focal minimal to mild neuronal degeneration in the cortex and focal minimal reduced layer of neuronal cells in the hippocampus (Figure 7C and Figure 8C). Moreover, treatment with quercetin 50 mg/kg showed multifocal minimal to mild neuronal degeneration at the cortex and hippocampus (Figure 7D and Figure 8D), additionally focal mild reduced layer of neuronal cells in the hippocampus. Quercetin, when administered with memantine at a dose of 20 + 25 mg/kg (Figure 7E and Figure 8E), showed multifocal minimal to mild neuronal degeneration at the cortex and hippocampus. The treatment group quercetin with memantine at a dose of 20 + 50 mg/kg (Figure 7F and Figure 8F) showed focal minimal neuronal degeneration at the cortex and hippocampus, which indicated an improvement in comparison to the disease control group. The arrow indicates the histopathological changes.

#### 2.4.2. Congo Red Staining

**Figure 9:**
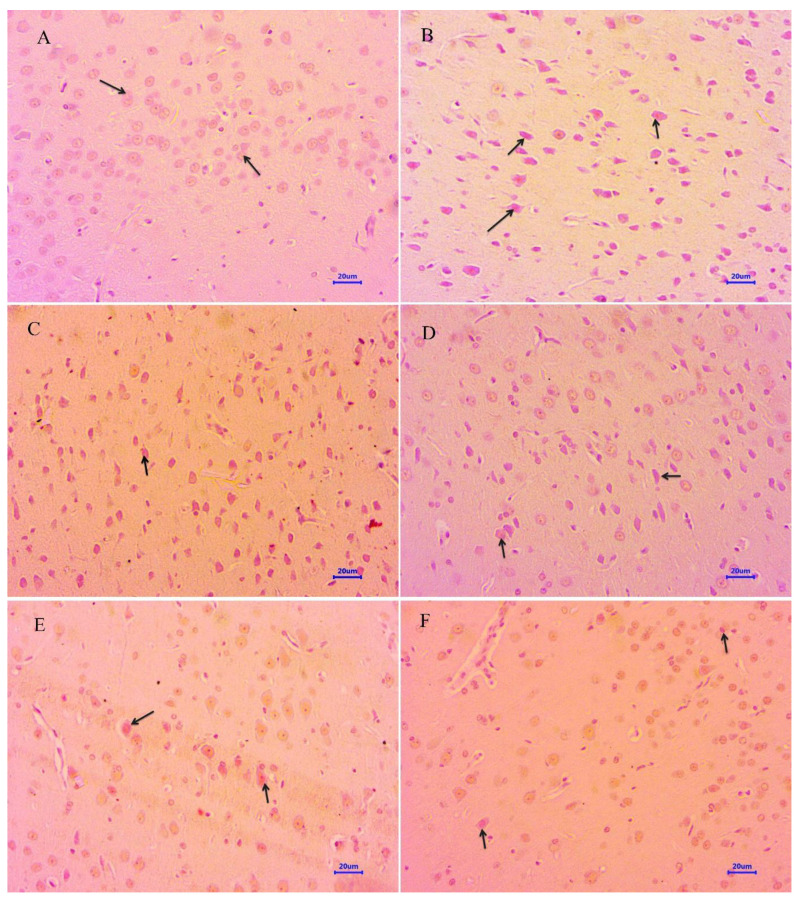
Effect of quercetin and quercetin administered with memantine on Congo red-stained cortex tissue (400×). (**A**): Normal control, (**B**): Disease control, (**C**): Memantine (20 mg/kg), (**D**): Quercetin (50 mg/kg), (**E**): Memantine + quercetin (20 + 25 mg/kg), (**F**): Memantine + quercetin (20 + 50 mg/kg).

**Figure 10:**
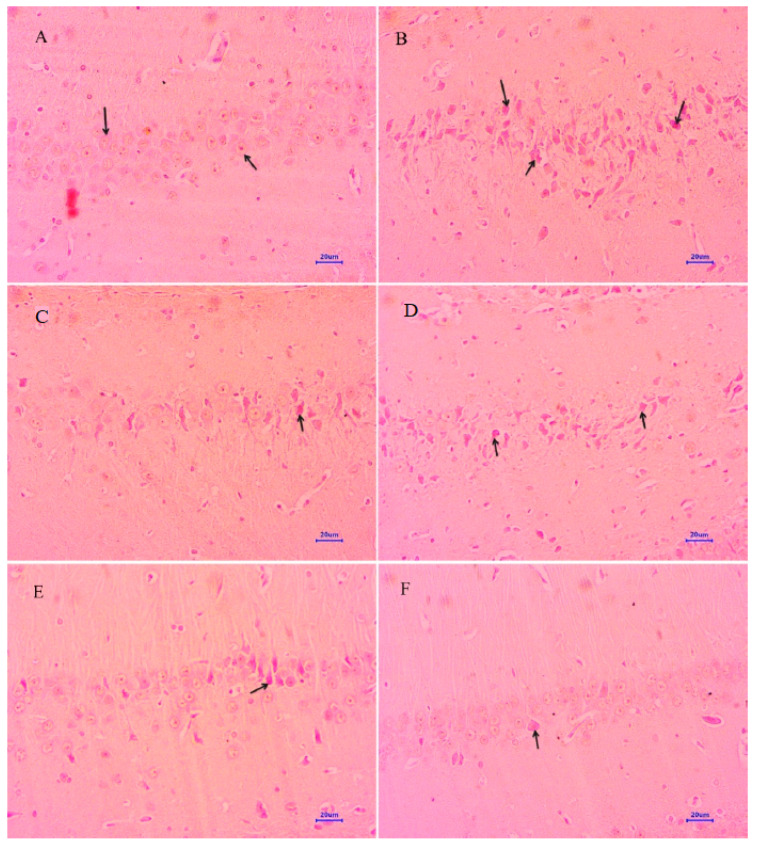
Effect of quercetin and quercetin administered with memantine on Congo red-stained hippocampus tissue (400×). (**A**): Normal control, (**B**): Disease control, (**C**): Memantine (20 mg/kg), (**D**): Quercetin (50 mg/kg), (**E**): Memantine + quercetin (20 + 25 mg/kg), (**F**): Memantine + quercetin (20 + 50 mg/kg).

Both cortex and hippocampus were stained using Congo red dye to detect the deposition of amyloid beta plaques. The cortex and hippocampus (Figure 9A and Figure 10A) from normal control group did not show any lesion of pathological significance. It showed normal histology and neuronal cells in the cortex and hippocampus. The brain tissues from disease control animals (Figure 9B and Figure 10B) showed deposition of amyloid in the cortex and hippocampus. Treatment with memantine 20 mg/kg (Figure 9C and Figure 10C) showed multifocal minimal to mild deposition of amyloid at the parietal cortex and hippocampus. The cortex and hippocampus (Figure 9D and Figure 10D) from the animals treated with quercetin (50 mg/kg) showed multifocal mild deposition of amyloid in the cortex as well as the hippocampus. Quercetin administered with memantine at a dose of 20 + 25 mg/kg (Figure 9E and Figure 10E) showed multifocal mild deposition of amyloid in the parietal cortex and hippocampus. However, the dose of 20 + 50 mg/kg of quercetin with memantine treatment showed focal minimal to mild deposition of amyloid in the cortex and hippocampus (Figure 9F and Figure 10F). The arrow indicates the histopathological changes.

### 2.5. Immunohistochemistry Studies (IHC)

#### 2.5.1. Amyloid-β Expression

**Figure 11:**
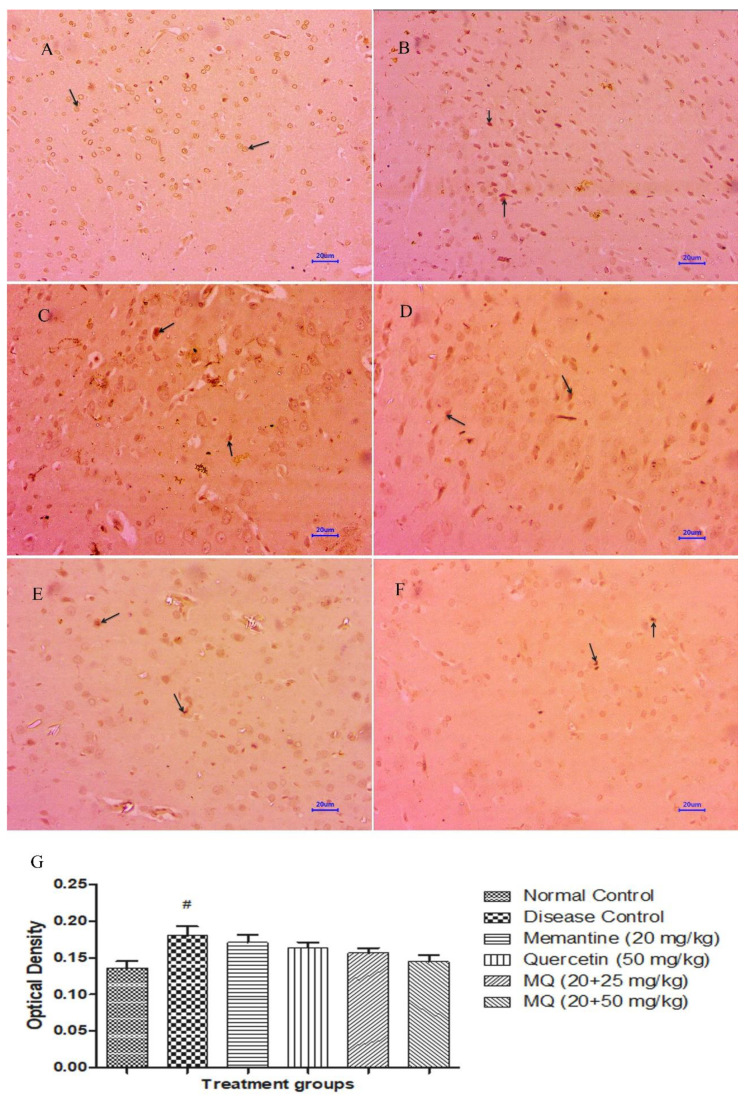
Effect of quercetin and quercetin administered with memantine on immuno-histochemical-stained cortex (400×). (**A**): Normal control, (**B**): Disease control, (**C**): Memantine (20 mg/kg), (**D**): Quercetin (50 mg/kg), (**E**): Memantine + quercetin (20 + 25 mg/kg), (**F**): Memantine + quercetin (20 + 50 mg/kg), (**G**): Optical density data. Data expressed as mean±SEM (n = 3), # *p* < 0.05 when compared with normal control.

**Figure 12:**
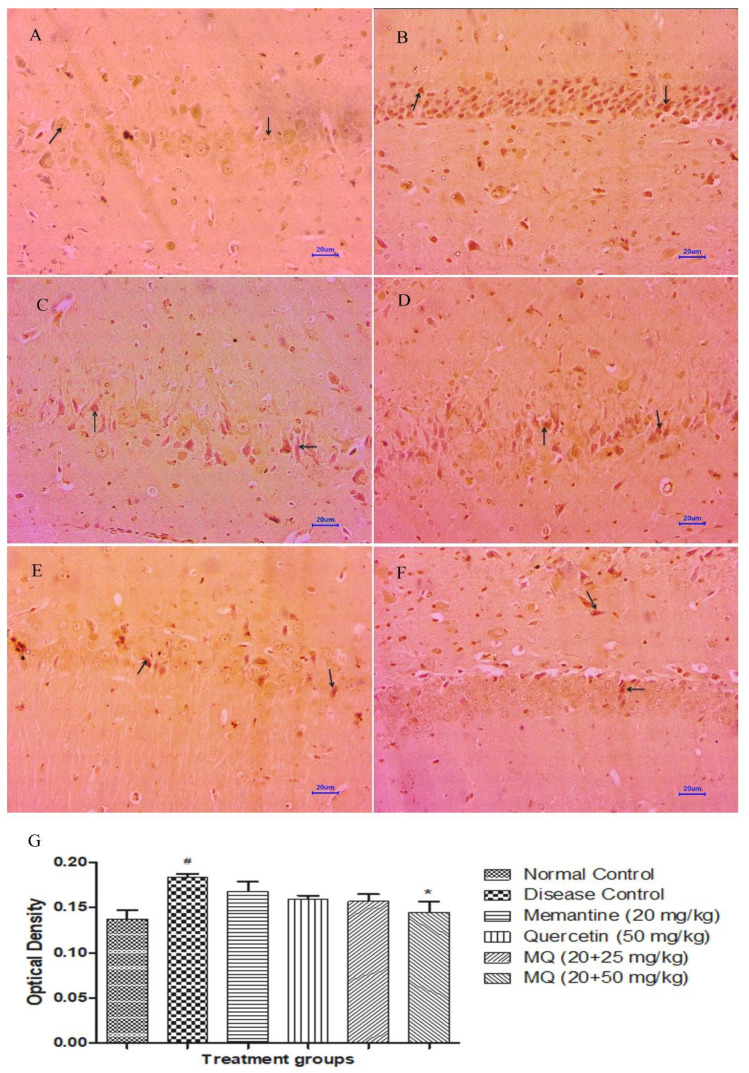
Effect of quercetin and quercetin administered with memantine on immuno-histochemical-stained hippocampus (400×). (**A**): Normal control, (**B**): Disease control, (**C**): Memantine (20 mg/kg), (**D**): Quercetin (50 mg/kg), (**E**): Memantine + quercetin (20 + 25 mg/kg), (**F**): Memantine + quercetin (20 + 50 mg/kg), (**G**): Optical density data. Data expressed as mean±SEM (n = 3), # *p* < 0.05 when compared with normal control group, * *p* < 0.05 when compared disease control group.

Microscopic examination of immuno-histochemical-stained brain tissues from the normal control group did not show expression of amyloid-β in cortex and hippocampus regions (Figure 11A and Figure 12A), while moderately enhanced expression of amyloid-β was observed in the cortex and hippocampus (Figure 11B and Figure 12B) of the disease control animals. The group administered with memantine 20 mg/kg showed mildly enhanced expression of amyloid-β in the cortex and hippocampus regions (Figure 11C and Figure 12C) of the brain. Further, the group administered with a dose of quercetin 50 mg/kg (Figure 11D and Figure 12D) and quercetin administered with memantine at a dose of 20 + 25 mg/kg (Figure 11E and Figure 12E) mildly enhanced expression of amyloid-β in cortex region and mild to moderately enhanced expression is observed in the hippocampus. However, the treatment of quercetin with memantine at a dose of 20 + 50 mg/kg (Figure 11F and Figure 12F) showed mildly enhanced expression of amyloid-β in both the cortex as well as hippocampus regions. Optical density data for IHC staining have also been presented, which confirms the microscopic observations (Figure 11G and Figure 12G).

The immunoreactivity was evaluated based on the percentage of positive neuronal cells, distribution of observed tissue section, and intensity of staining and was graded as No reactivity, minimal, mild, moderate, marked/moderately severe, and severe.

#### 2.5.2. BDNF Expression

**Figure 13:**
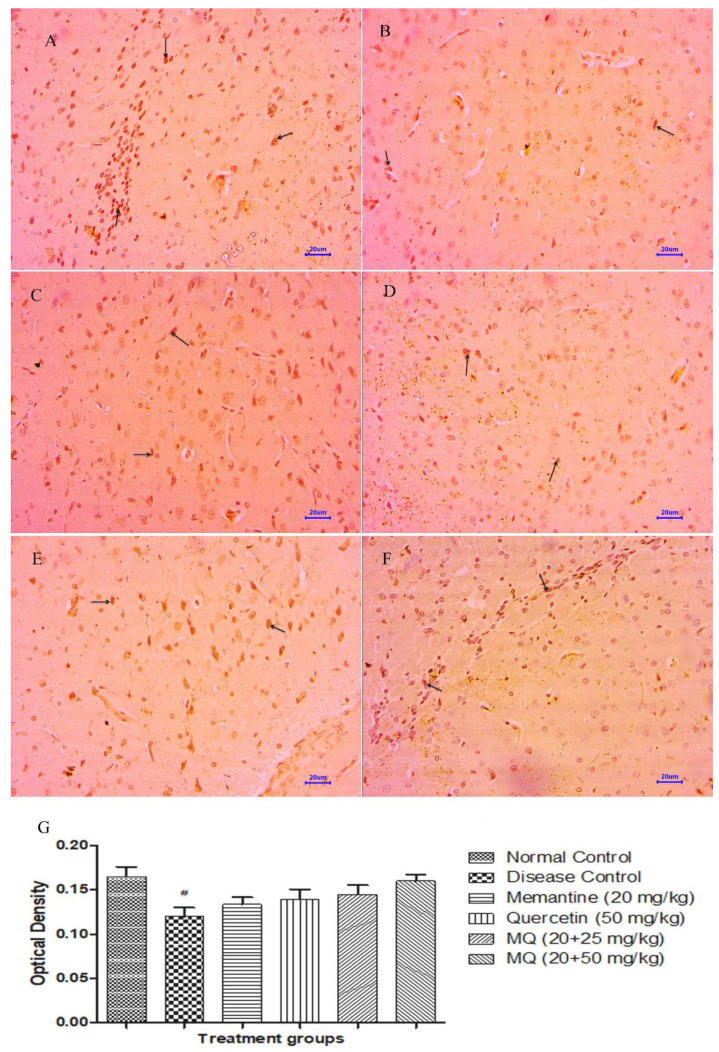
Effect of quercetin and quercetin administered with memantine in BDNF expression in cortex. (**A**): Normal control, (**B**): Disease control, (**C**): Memantine (20 mg/kg), (**D**): Quercetin (50 mg/kg), (**E**): Memantine + quercetin (20 + 25 mg/kg), (**F**): Memantine + quercetin (20 + 50 mg/kg), (**G**): Optical density data. Data expressed as mean±SEM (n = 3), # *p* < 0.05 when compared with normal control group.

**Figure 14:**
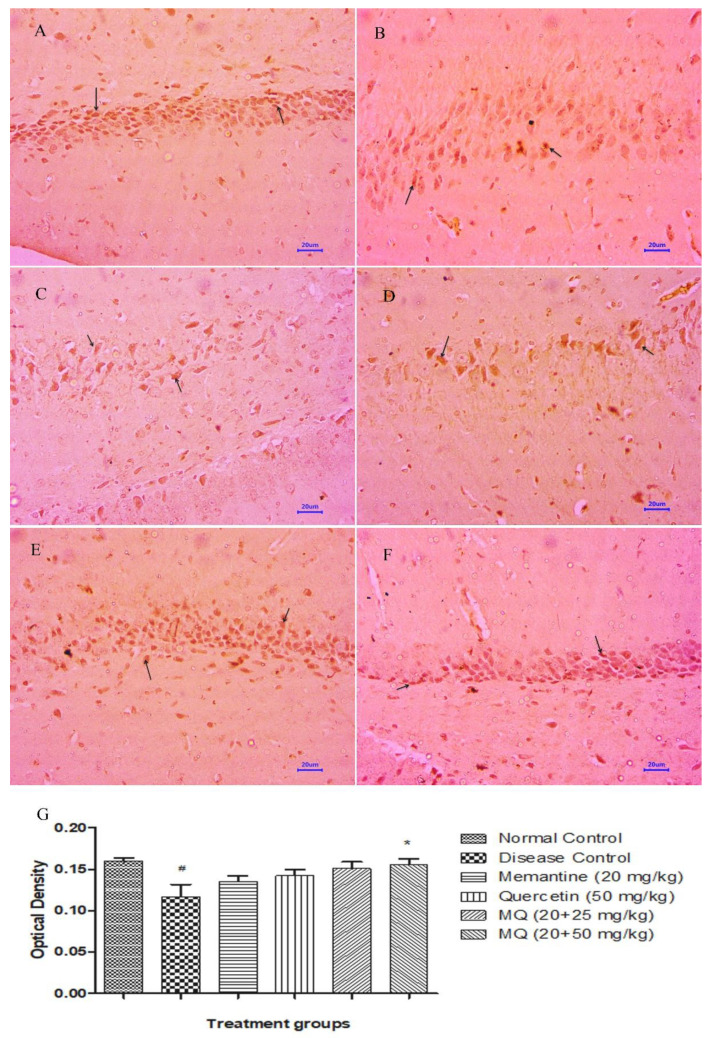
Effect of quercetin and quercetin administered with memantine in BDNF expression in hippocampus. (**A**): Normal control, (**B**): Disease control, (**C**): Memantine (20 mg/kg), (**D**): Quercetin (50 mg/kg), (**E**): Memantine + quercetin (20 + 25 mg/kg), (**F**): Memantine + quercetin (20 + 50 mg/kg), (**G**): Optical density data. Data expressed as mean±SEM (n = 3), # *p* < 0.05 when compared with normal control group, * *p* < 0.05 when compared disease control group.

Immunohistochemical (IHC) assessment for the expression of BDNF in the tissue samples of the cortex and hippocampus (Figure 13B and Figure 14B) region showed mild expression of BDNF in disease control animals, whereas brain samples from normal control animals have shown moderately enhanced expression of BDNF in cortex and hippocampus (Figure 13A and Figure 14A) regions of the brain. Treatment with memantine 20 mg/kg (Figure 13C and Figure 14C) showed mild to moderately enhanced expression of BDNF in the cortex and hippocampus. Moreover, the treatment group administered with quercetin 50 mg/kg indicated mild to moderately enhanced expression in the cortex and mild expression of BDNF in the hippocampus (Figure 13D and Figure 14D). However, the treatment group administered with quercetin and memantine 20 + 25 mg/kg (Figure 13E and Figure 14E) and 20 + 50 mg/kg (Figure 13F and Figure 14F) showed mild to moderately enhanced expression of BDNF in the cortex and hippocampus of the brain. Optical density data for IHC staining have also been presented, which confirms the microscopic observations. (Figure 13G and Figure 14G).

The immunoreactivity was evaluated based on the percentage of positive neuronal cells, distribution of observed tissue section, and intensity of staining and was graded as no reactivity, minimal, mild, moderate, marked/moderately severe, and severe.

## 3. Discussion

In the last decade, a tremendous amount of effort has been made to understand the multifactorial etiology of Alzheimer’s disease [21]. The most likely risk factors include age and genetics, while others may include head injury, exposure to toxins or heavy metals, trisomy, sex, and cardiovascular diseases [4,21,22].

Aluminum (Al) is a potent neurotoxin, and prolonged exposure to it has been linked with pathophysiological changes and an increase in oxidative stress, which in turn affects various cognitive functions [23]. It has also been observed that chronic administration of aluminum leads to the depletion of AChE and degeneration of cholinergic fibers in the cortex and hippocampus region of the brain [24]. It has been reported that aluminum administration increases the expression of Aβ (1–42), β and γ- secretases in the hippocampus and cortex of rats, suggesting that Al toxicity leads to the Aβ formation [25,26,27]. Further, it has also been observed that aluminum encourages the foundation for the tau-NFT cascade in motion by inhibiting protein phosphatase 2A (PP2A) activity, which is fundamental to AD. Therefore, we decided to use an animal model of aluminum chloride (AlCl_3_)-induced neurotoxicity in this study.

Research suggests that a combination of drugs that can target multiple aspects of AD pathology can be more effective in the treatment of AD [28]. Based on the available literature, memantine addresses dysfunction in glutamatergic transmission, while quercetin has the capacity to quench oxygen free radicals, suppressing the lipid peroxidation, altering the redox status of glutathione and inhibiting acetylcholinesterase effect [17,29,30,31]. Therefore, we devised a plan to study the effect of quercetin with memantine in the aluminum chloride (AlCl_3_)-induced neurotoxicity in rats.

In the present study, aluminum chloride was administered by oral route manifesting oxidative stress leading to progressive deterioration of neurons resulting in cognitive impairment and memory loss. The study result confirmed the same. To study the effect of combination treatment, locomotor activity and various other behavior models such as MWM, EPM, and PA were assessed during the present study.

In the β-amyloid precursor protein-deficient mice, significant impairment in locomotor activity was reported indicating a compromised neuronal or muscular function [32]. Treatment with quercetin and memantine has significantly improved locomotor activity in animals when compared with the disease control group.

The result from the MWM indicated that administration of quercetin with memantine significantly decreased escape latency as compared to the disease control group indicating improvement in learning and memory skills.

The data from the EPM study showed significant decrease in the retention transfer latency in disease control group as compared to the normal control group. On the contrary, quercetin with memantine facilitated retention of memory when compared with the AlCl_3_-treated group.

In the present study, the retention of memory was also tested by measuring pre- and post-shock latency in the passive avoidance (PA) test. The disease control animals have shown a decline in post-shock latency when compared with the normal control group. By contrast, a significant increase in post-shock latency has been observed in the treatment group of quercetin with memantine as compared to the disease control group.

An increase in oxidative stress is a result of an imbalance between free radicals and scavenging activity. During normal physiological conditions, various enzymes such as superoxide dismutase, catalase, glutathione peroxidase, and glutathione reductase ameliorate the impact of free radicals for the protection of intracellular processes and organelles [33]. In this study, we evaluated various oxidative stress parameters, and the result from the study demonstrated that quercetin with the memantine-treated group significantly inhibited oxidative damage by scavenging free radicals. Quercetin has been reported to inhibit nuclear factor kappa-B, reducing the expression of pro-inflammatory matrix metalloproteases and elevating nitric oxide levels. Further, it has been reported that through nicotinamide adenine dinucleotide phosphate oxidase production, quercetin can restore mitochondrial functions [34,35].

Acetylcholinesterase inhibitors (AChEIs) are used for the treatment of AD. Acetylcholineestarase (AChE) inhibitors are used to restore the normal cholinergic function in the patients of AD [36]. In the present study, increase in AChE activity was observed in the AlCl_3_-treated group as compared to normal control group. On the contrary, the quercetin-treated group indicated a significant inhibitory effect as compared to the disease control group.

Microscopic examination of the cortex and hippocampus stained with hematoxylin and eosin (H&E) showed various lesions among different groups. Examination of the cortex and hippocampus of rats from the normal group did not show any lesion of pathological significance. By contrast, brain tissues from the disease control group showed multifocal moderate neuronal degeneration at the cortex and hippocampus. Animals treated with quercetin and memantine showed focal minimal neuronal degeneration at the cortex and hippocampus. It demonstrated that treatment of quercetin with memantine at a dose of 20 + 50 mg/kg had reversed the neurotoxic effect of the AlCl_3_ due to its neuroprotective activity. The extent of deposition of Aβ plaque in the brain was assessed using Congo red stain, and it was observed multifocal moderate deposition of amyloid-β in the cortex and hippocampus of disease control animals, which were absent in the normal control group. The treatment of quercetin with memantine at a dose of 20 + 50 mg/kg showed minimal to mild deposition of amyloid-β at the cortex and hippocampus, indicating that it is effective in inhibiting the expression.

We also studied the expression of BDNF by microscopic examination through immuno-histochemical staining. The result from the present study indicated that quercetin with memantine at a dose of 20 + 50 mg/kg showed mild to moderately enhanced expression of BDNF in the cortex region and moderately enhanced expression in the hippocampus as compared to the disease control group.

In many studies, it has been reported that amyloid-β inhibits hippocampal long-term potentiation (LTP), which transiently impairs learning, memory, and behavior in rats [37]. In addition to reducing NMDAR functions, amyloid-β promotes endocytosis of NMDARs, long-term depression (LTD), inhibition of PKC-mediated mGluR activation of NMDARs and GABARs, which results in the impairment of memory and learning [38]. Therefore, considering the onset of deficits in glutamatergic synaptic transmission, memantine has been proven to be beneficial for AD therapy.

## 4. Material and Methods

### 4.1. Drugs and Chemicals

Acetylthiocholine iodide, AlCl_3,_ and quercetin were purchased from Sigma Aldrich (St. Louis, MO, USA), and memantine was a gift sample received from Intas Pharmaceuticals Limited, Ahmedabad, India. β-Amyloid (B4) antibody, goat anti-rabbit IgG antibody, and pro-BDNF antibodies were procured from SantaCruz Biotechnology Inc., USA. All other chemicals used in the study were of analytical grade.

### 4.2. Animals

Male albino Wistar rats (180–220 g) were procured from the National Institute of Biosciences, Pune, India. They were housed in a group of 5 in a cage at a room temperature of 25 ± 2 °C and relative humidity of 50 ± 10%. Animals were kept under 12 hr of light-dark cycle for at least 7 days before the start of an experiment. During the whole experiment, animals were given access to a standard diet and water ad libitum. All procedures regarding animal care and use were followed based on the regulations dictated by the Animal Ethics Committee. The experimental protocol was approved by the Institutional Animal Ethics Committee, which is working in accordance with the requirements of the purpose of control and supervision of experiments on animals (CPCSEA), the government of India, and also comply with guidelines laid down by the National Institute of Health (NIH) for the handling of experimental animals.

### 4.3. Experimental Design

A total of 60 albino Wistar rats (180–220 gm) were randomized based on their body weight into 6 groups (10 animals per group): (1) normal control group was administered with distilled water; (2) disease control group received only AlCl_3_ (100 mg/kg) orally; (3) memantine (20 mg/kg) orally; (4) received oral quercetin 50 mg/kg orally; (5) memantine and quercetin 20 + 25 mg/kg orally; and (6) memantine and quercetin 20 + 50 mg/kg orally

Except for animals from the normal control group, all other animals were administered with oral AlCl_3_ (100 mg/kg) dissolved in distilled water for 42 days [39].

### 4.4. Behavioral Assessment

#### 4.4.1. Actophotometer

The locomotor activity of each animal was observed on days 21 and 42 from the start of the administration of AlCl_3_ (100 mg/kg p.o.). Animals were kept in the actophotometer cubical for 5 mins, which is equipped with infrared light-sensitive photocells. Every time the beam of light falling on the photocell is cut off due to the movement of an animal, it is recorded as one count [39].

#### 4.4.2. Morris Water Maze

Morris water maze (MWM) is behavior assessment, performed to evaluate spatial learning and memory in rats. We conducted the tests with slight modifications to the procedures laid down by Morris Richard [39,40,41]. A circular pool of water having 150 cm of diameter and 45 cm of height was used for evaluation. The tank was filled with water having temp 25 ± 2 °C. Further, the tank was marked to divide it in four equal quadrants, which were kept unchanged throughout the study and a round platform measuring 10 cm X 10 cm was kept in one of the quadrants.

The fundamental feature of the technique is that rats are placed in the tank facing toward the wall of the tank and allowed to find the platform. The experiment was conducted in two phases; an acquisition phase was conducted for 4 consecutive days from the 17th to the 20th day, whereas the retention phase was conducted on days 21 and 42. During the acquisition phase, animals were placed in the tank and were allowed to locate the platform, which was kept 1 cm above the water level. If the animal could not locate the platform within 120 sec, it was guided to reach the platform and allowed to stay for 30 sec on the platform. Every day, four trials were given to each animal at an interval of 10 min between two trials, and each time animal was placed in a different quadrant.

During the retention phase, on days 21 and 42, the platform was hidden under the water by making the water opaque. Thus, the platform offers no clue to escape behavior.

In principle, animals could find a platform by swimming arbitrarily throughout the pool; but after the acquisition phase, normal animals learned quickly to swim straight toward the platform. The quick escape toward the platform indicates evidence of spatial learning and memory.

#### 4.4.3. Elevated Plus Maze

Elevated plus maze (EPM) is also used to study the effect of drugs on learning and memory by evaluating transfer latency (TL) in rats [39,42,43].

The EPM apparatus comprises two open and two enclosed arms connected with a central square of 10 × 10 cm. The arms are about 50 cm long, 10 cm wide, and elevated about 50 cm from the ground.

The assessment was performed in two phases’ acquisition and phase trials. In an acquisition trial that was performed on day 20th, initial transfer latency (ITL) was recorded as the time required for the animal to move from open arm to closed arm. After entering the closed arm, the animal was allowed to explore for 20 sec inside the maze and then was returned to the home cage. Retention of memory was evaluated on days 21 and 42 as 1st transfer latency and 2nd transfer latency, respectively.

#### 4.4.4. Passive Avoidance

The passive avoidance (PA) test determines the ability of an animal to assess memory retention deficit [44]. The device consisted of two compartments (25 × 25 × 25 cm): one was brightly illuminated, whereas the other one was dark. The bottom of the dark compartment had a metal grid attached to an electric source supply. This assessment was also performed in two phases: acquisition and retention.

During an acquisition phase, the animal was placed in a bright compartment for 60 s, after which the separating door was opened, and the time taken by the animal to step into the dark compartment was recorded as latency. After entering the dark compartment, a mild electric shock (0.5 mA for 2 s) was delivered through the metal grid to the animal. After the shock, the animal was taken out from the dark compartment and returned to the home cage to form a link between the dark compartment and the foot shock. The retention trial was performed on days 21 and 42.

### 4.5. Biochemical Assessment

#### 4.5.1. Brain Tissues

Animals were euthanatized using CO_2_ asphyxiation following the completion of behavioral assessment. Brains were removed and washed with an ice-cold isotonic normal saline. Further, the cortex and hippocampus were separated and homogenized with 10% (*w*/*v*) of 0.1 M phosphate buffer (pH 7.4) using a probe homogenizer (Polytron PT 2500E, Kinematica, Malters, Switzerland). The homogenized tissue samples were used for the assessment of biochemical parameters.

#### 4.5.2. Oxidative Stress Parameters

The protein content in the homogenized tissue was analyzed as per the method described by Lowry et al. [45]. The estimation for malondialdehyde (MDA) was carried out using tissue homogenate as per the method described by Ohkawa et al. [46]. Further, the activity of reduced glutathione was performed using tissue homogenate defined by the method of Ellman [47]. The method defined by Paoletti et al. was used for the estimation of superoxide dismutase (SOD) using post-mitochondrial supernatant (PMS) [48,49]. To obtain post-mitochondrial supernatant (PMS), the tissue homogenate was centrifuged at 10,000 rpm for 20 min at 4 °C. The estimation of catalysis was performed by the method of Lück using post-nuclear supernatant (PNS), which was prepared by centrifuging tissue homogenate at 2500 rpm for 10 min at 4 °C.

#### 4.5.3. Acetylcholinesterase Activity (AChE)

The analysis of acetylcholinesterase (AChE) was performed as per the method of Ellman [50]. AChE is an indicator of the degeneration of cholinergic neurons. The assay mixture contained 0.1 mL of supernatant, 2 mL of sodium phosphate buffer (0.1M, pH 8.0), 0.1% BSA solution, and 0.1 mL of DTNB (Ellman regent). The change in absorbance was measured over 2 min at an interval of 1 min using a UV-Vis spectrophotometer (Perkin Elmer Lambda 20, Waltham, MA, USA) at 412 nm of wavelength. The difference in the intensity of color was measured, and the results of the assay were expressed as micromoles of acetyl thiocholine iodide hydrolyzed/min/mg of protein.

### 4.6. Histopathological Assessment

For histopathological evaluation, the brains removed were immediately placed in 10% neutral buffered formalin (NBF). From each treatment group, 3 slides were examined as a part of the histopathology study for both hippocampus and cortex. The hippocampus or cortex tissues were fixed on a paraffin block, and transverse sections of 4–6 µm thickness were sliced using a rotary microtome (Leica, New York, NY, USA). The sections were stained with hematoxylin and eosin (H&E) and Congo red and were examined using a digital microscope (Motic, Richmond, BC, Canada) [51,52,53,54]. The severity of neurodegeneration was evaluated under 400X magnification [25,51]. The scoring of neurodegeneration was graded on the scale of observed lesions recorded as Not Present, Minimal (<1%), Mild (1–25%), Moderate (26–50%), Marked/Moderately Severe (51–75%), and Severe (76–100%). Further, the distribution of neurodegeneration was also recorded as focal, multifocal, and diffused.

### 4.7. Immunohistochemical Assessment

Immunohistochemical (IHC) assessments of hippocampus and cortex tissue for expression of amyloid-β and BDNF were performed. The paraffin wax embedded tissue blocks were sectioned at 4–6 µm thickness with the rotary microtome (Leica, USA) and placed on slides coated with Poly-L-Lysine and placed in an incubator overnight at 37 °C. Further, these sections were deparaffinized, rehydrated, and incubated with citrate buffer, pH 6, in the decloaking chamber. Slides were incubated in a 3% hydrogen peroxide block for 20 min to block endogenous peroxidase. Human anti-rat β-Amyloid (b-4) antibody was applied as the primary antibody, and peroxidase-labeled goat-anti-rabbit IgG as the secondary antibody. The staining was visualized by reaction with diaminobenzidine color reagent and then counterstained with hematoxylin. Finally, the sections were rinsed with Tris buffer saline (TBS) and dehydrated in alcohol, and cleared in xylene prior to mounting using DPX. All the sections were examined by the light microscope to record the intensity of the antigen-antibody reaction [51,55].

For immunohistochemical assessment for the expression of BDNF (pro-BDNF:5H8) primary antibodies, a similar procedure was followed [55].

The immunoreactivity in the present study was evaluated based on the percentage of positive neuronal cells, distribution of observed tissue section, and intensity of staining and was graded (Table 3). 

### 4.8. Statistical Analysis

All the data collected during the study were statistically evaluated using GraphPad Prism V 5.0. The statistical analysis for the behavioral studies was performed using two-way ANOVA (analysis of variance) at a significance level of 0.05. Bonferroni’s test was applied to determine the significance between groups. The data from the oxidative stress parameters and acetylcholinesterase activity were evaluated using one-way ANOVA at a 0.05 level of significance. Dunnett’s multiple comparison test was used for the comparison. All the data are expressed as mean ± standard error (mean ± SEM). The optical density of the IHC images was analyzed using ImageJ 1.8.0 software.

## 5. Conclusions

The aim of the present study was to investigate the effect of quercetin with memantine against AlCl_3-_induced oxidative stress, memory degradation, and neuronal cell death. It has been reported that the administration of AlCl_3_ is responsible for oxidative stress, which leads to the peroxidation of lipid membranes and cell apoptosis. Further, the dysfunction of neuronal cells and progressive death of neurons are also associated with oxidative stress [19,56].

From the results, it can be indicated that the administration of quercetin with memantine has significantly improved behavior parameters, learning, and spatial memory. The administration of quercetin with memantine has shown significant improvement in GSH, catalase, and SOD levels and a decrease in MDA levels. It is an indication of a reduction in the oxidative stress parameters, which are critical to neurodegeneration or mitochondrial damage. The ability of quercetin to inhibit oxidative stress comes from the property of quenching reactive oxygen species. Quercetin has also shown significant acetylcholinesterase inhibitory activity. Inhibition of amyloid-β plaque formation and increase in the levels of BDNF in the brain tissues of animals treated with quercetin and memantine signaling synergistic treatment effect.

The study results indicated that quercetin with memantine protected the degradation of learning and loss of memory.

In conclusion, the data from the study indicates that quercetin and memantine treatment has significant neuroprotective effects in AlCl_3_ induced neurotoxicity. However, to confirm the clinical benefits of the quercetin and memantine combination, more studies are required to be performed.

## Figures and Tables

**Table 1:** Effect of quercetin and quercetin with memantine treatment on oxidative stress parameters in the cortex.

Groups	MDA (nmol/mg of Protein)	SOD (µmol/mg of Protein)	CAT (nmol of H_2_O_2_ Decomposed/min mg of Protein)	GSH (µmol/mg of Protein)
Normal control	3.78 ± 0.33	8.48 ± 0.48	8.32 ± 0.40	10.42 ± 0.48
Disease control	7.38 ± 0.36 ^###^	5.13 ± 0.34 ^###^	3.73 ± 0.09 ^###^	6.07 ± 0.40 ^###^
Memantine (20 mg/kg)	6.57 ± 0.46	5.68 ± 0.54	4.78 ± 0.30 **	7.56 ± 0.67 *
Quercetin (50 mg/kg)	5.22 ± 0.24 ***	5.99 ± 0.64	5.12 ± 0.28 **	7.69 ± 0.37 *
Memantine + Quercetin (20 + 25 mg/kg)	4.42 ± 0.35 ***	7.22 ± 0.45*	6.09 ± 0.18 ***	8.56 ± 0.39 **
Memantine + Quercetin (20 + 50 mg/kg)	4.08 ± 0.29 ***	7.75 ± 0.46**	7.87 ± 0.18 ***	9.17 ± 0.40 ***

Data expressed as mean ± SEM (n = 6), ^###^ *p* < 0.001 when compared with the normal control group, * *p* < 0.05, ** *p* < 0.01, *** *p* < 0.001 when compared with the disease control group. SEM: standard error of mean.

**Table 2:** Effect of quercetin and quercetin with memantine treatment on oxidative stress parameters in the hippocampus.

Groups	MDA (nmol/mg of Protein)	SOD (µmol/mg of Protein)	CAT (nmol of H_2_O_2_ Decomposed/min mg of Protein)	GSH (µmol/mg of Protein)
Normal control	3.91 ± 0.27	8.10 ± 0.42	8.35 ± 0.48	10.52 ± 051
Disease control	7.19 ± 0.24 ^###^	4.43 ± 0.62 ^###^	4.17 ± 0.32 ^###^	4.99 ± 0.59 ^###^
Memantine (20 mg/kg)	6.56 ± 0.53	4.97 ± 0.28	4.54 ± 0.48	5.93 ± 0.55
Quercetin (50 mg/kg)	5.18 ± 0.35 **	6.98 ± 0.61 **	6.36 ± 0.55 *	7.77 ± 0.60 **
Memantine + Quercetin (20 + 25 mg/kg)	4.42 ± 0.43 ***	7.43 ± 0.52 ***	7.06 ± 0.52 **	8.59 ± 0.44 ***
Memantine + Quercetin (20 + 50 mg/kg)	4.10± 0.38 ***	7.75 ± 0.45 ***	7.70 ± 0.59 ***	9.28 ± 0.48 ***

Data expressed as mean ± SEM (n = 6), ^###^ *p* < 0.001 when compared with normal control group, * *p* < 0.05, ** *p* < 0.01, *** *p* < 0.001 when compared with the disease control group. SEM: standard error of mean.

**Table 3:** The Grading of Immunoreactivity.

Percentage of Positive Cells	Intensity of Staining
0	No reactivity
<1%	Minimal
1–25%	Mild
26–50%	Moderate
51–75%	Marked/moderately severe
76–100%	Severe

## Data Availability

Data sharing is not applicable to this article.

## Sample Availability

Samples of the compounds are available from the authors.
